# Thirty-Day Postdischarge Mortality Among Black and White Patients 65 Years and Older in the Medicare Hospital Readmissions Reduction Program

**DOI:** 10.1001/jamanetworkopen.2019.0634

**Published:** 2019-03-15

**Authors:** Peter Huckfeldt, José Escarce, Neeraj Sood, Zhiyou Yang, Ioana Popescu, Teryl Nuckols

**Affiliations:** 1 University of Minnesota School of Public Health, Minneapolis; 2 Division of General Internal Medicine and Health Services Research, David Geffen School of Medicine at UCLA, Los Angeles, California; 3 Sol Price School of Public Policy, University of Southern California, Los Angeles; 4 Division of General Internal Medicine, Cedars-Sinai Medical Center, Los Angeles, California

## Abstract

**Importance:**

The Medicare Hospital Readmissions Reduction Program (HRRP) has disproportionately penalized hospitals that treat many black patients, which could worsen health outcomes in this population.

**Objective:**

To determine whether short-term mortality rates increased among black and white adults 65 years and older after initiation of the HRRP and whether trends differed by race.

**Design, Setting, and Participants:**

In a cohort study using an interrupted time-series analysis conducted from March 15, 2018, to January 23, 2019, in 3263 eligible acute care hospitals nationally, risk-adjusted mortality rates observed after Medicare started to impose penalties (October 1, 2012, to November 30, 2014) were compared with projections based on pre-HRRP trends (January 1, 2007, to March 31, 2010) among adults 65 years and older with acute myocardial infarction (AMI), heart failure (HF), or pneumonia. Observed-to-projected differences were then compared between racial groups.

**Exposures:**

Hospital discharge during pre-HRRP and HRRP penalty periods.

**Main Outcomes and Measures:**

Thirty-day postdischarge all-cause mortality.

**Results:**

In the 3263 acute care hospitals included in the analysis, black patients (627 373 index discharges) were more likely than white patients (5 845 130 index discharges) to be younger (mean [SD] age, 77.8 [8.3] vs 80.5 [8.2] years; *P* < .001), women (60.5% vs 53.7%; *P* < .001), dually covered by Medicare and Medicaid (45.7% vs 17.2%; *P* < .001), and treated at a penalized hospital (AMI, 82.8%; HF, 83.8%; and pneumonia, 82.6% vs 69.6%; 73.3%; and 71.7%; all *P* < .001). Pre-HRRP mortality rates for black vs white patients were 7.04% (95% CI, 6.75% to 7.33%) vs 7.47% (95% CI, 7.37% to 7.57%) for AMI, 6.69% (95% CI, 6.56% to 6.82%) vs 8.56% (95% CI, 8.48% to 8.64%) for HF, and 8.08% (95% CI, 7.88% to 8.27%) vs 8.27% (95% CI, 8.19% to 8.35%) for pneumonia. By the HRRP penalty period, observed mortality for AMI decreased more, relative to projections, among black than white patients (difference-in-differences, −1.65 percentage points; 95% CI, −3.19 to −0.10; *P* = .04). For HF, mortality increased relative to projections among white patients but not among black patients; however; mortality trends did not differ by race (difference-in-differences, −0.37 percentage points; 95% CI, −1.08 to 0.34; *P* = .31). For pneumonia, observed mortality was similar to projections in both racial groups, and trends did not differ by race (difference-in-differences, −0.54 percentage points; 95% CI, −1.66 to 0.59; *P* = .35). At both penalized and nonpenalized hospitals, mortality trends were similar or decreased more among black patients than white patients.

**Conclusions and Relevance:**

In this study of patients 65 years and older, short-term postdischarge mortality did not appear to increase for black patients under the HRRP, suggesting that certain value-based payment policies can be implemented without harming black populations. However, mortality seemed to increase for white patients with HF and this situation warrants investigation.

## Key Points

**Question:**

In the Medicare Hospital Readmissions Reduction Program, did mortality from all causes increase during the 30 days after hospital discharge among black vs white patients 65 years and older?

**Findings:**

In a cohort study using a time-series analysis including 3263 acute care hospitals, short-term mortality decreased more among black patients than white patients with acute myocardial infarction. Mortality increased among white patients with heart failure, but trends over time did not differ between black and white patients; and mortality trends over time were stable and similar between black and white patients with pneumonia.

**Meaning:**

This study suggests that value-based payment policy was not associated with an increase in mortality among black populations, but causes of increasing mortality among white patients warrant investigation.

## Teaser

This cohort study uses a time-series analysis to examine differences in mortality and hospital readmissions in black vs white patients 65 years and older after introduction of the Medicare Hospital Readmissions Reduction Program.

## Introduction

Health care payers are implementing value-based payment models that incentivize performance on measures of quality and cost.^1,2^ However, the National Academy of Medicine and others have expressed concern that such policies disproportionately penalize health care institutions that care for vulnerable populations including racial minorities. By taking funds away from institutions with greater resource needs, these policies may promote socioeconomic disparities in health outcomes.^3,4,5,6^

One substantive foray into value-based payment is the Medicare Hospital Readmissions Reduction Program (HRRP), established in March 2010 and implemented in October 2012. This policy penalizes acute care hospitals with elevated 30-day unplanned readmission rates among fee-for-service Medicare beneficiaries 65 years and older who are admitted with acute myocardial infarction (AMI), heart failure (HF), or pneumonia.^7^ Since 2010, readmissions have decreased, particularly at penalized hospitals.^8,9,10^ Notably, some studies suggest that 30-day postdischarge mortality rates have risen among adults 65 years and older with HF and pneumonia.^11,12,13,14,15^

Historically, black adults 65 years and older with these 3 conditions (black patients) had higher risk-adjusted 30-day readmission and lower risk-adjusted 30-day mortality rates than white adults 65 years and older (white patients).^16,17^ Yet there are several reasons that mortality may have increased more among black patients than white patients under the HRRP. First, hospitals with the largest proportions of black patients have incurred more penalties,^10^ reducing the resources available for care. Health care for black patients is concentrated at smaller hospitals that treat many Medicaid recipients, perform poorly on quality measures, and have elevated mortality rates.^18,19,20^ Until 2018, Medicare assigned HRRP penalties without accounting for patients’ socioeconomic characteristics.^7^ Second, even within the same hospitals, the policy may have disproportionately affected black patients because of their higher readmission rates. Readmissions have decreased more among black patients than white patients,^10,21^ which could owe to better transition-related care or barriers to rehospitalization. Third, any barriers to readmission could pose greater risks of harm for black patients, who often face worse challenges to managing acute illnesses at home because of higher rates of poverty and poorer access to outpatient care.^17,22,23,24,25^

Accordingly, we tested the a priori hypotheses that 30-day postdischarge mortality increased among black patients with AMI, HF, and pneumonia, and that increases in mortality were greater among black than white patients treated at the same hospitals. We also compared trends in 30-day readmissions. We studied each target condition separately because mortality trends have differed across them.^11^ In addition, because penalized hospitals treated more black patients and faced greater pressure to reduce readmissions, we stratified secondary analyses by hospital penalty status.

## Methods

From March 15, 2018, to January 23, 2019, we performed an interrupted time-series analysis of 30-day mortality and readmission rates among black and white patients admitted with AMI, HF, or pneumonia in a pre-HRRP period (January 1, 2007, to March 31, 2010), an anticipation period (April 1, 2010, to September 30, 2012), and a penalty period (October 1, 2012 to November 30, 2014). This study followed the Strengthening the Reporting of Observational Studies in Epidemiology (STROBE) reporting guideline.^26^ Institutional review boards at the University of California, Los Angeles; Cedars-Sinai Medical; the University of Southern California; and the University of Minnesota approved this analysis and waived informed consent because data were deidentified.

### Setting and Population

Study hospitals were eligible for the HRRP in fiscal years 2013 or 2014. Penalty status was based on fiscal year 2013. Participants included black and white Medicare fee-for-service beneficiaries 65 years or older with an eligible index discharge from a study hospital between January 1, 2007, through November 30, 2014, and a principal diagnosis of AMI, HF, or pneumonia based on *International Classification of Diseases, Ninth Revision, Clinical Modification* codes used under the policy. As per the HRRP, exclusion criteria included death during hospitalization, discharge to another hospital, release against medical advice, hospitalization lasting over 365 days, discharge for the same condition in the preceding 30 days, and discontinuous Medicare Part A and B enrollment in the year before or month after the index hospitalization.^7^ The unit of analysis was the index hospitalization and 30-day postdischarge period.^7,27^

### Data Sources

We obtained the principal diagnosis, comorbidities, and discharge disposition from the 2007-2014 Medicare Provider and Analysis Review files. Medicare enrollment, dual Medicaid enrollment, age, race, sex, and mortality came from the 2005-2014 Master Beneficiary Summary Files. In these data, race was based on patient self-report.

Hospital eligibility and penalty status was based on the Medicare HRRP payment adjustment factor data sets for fiscal year 2013-2014.^7^ Other hospital characteristics were from the 2012 Medicare Provider of Services files.

### Measures

The principal outcome measure was risk-adjusted, 30-day all-cause mortality, meaning death within 30 days after discharge alive from an index hospitalization. A secondary outcome was risk-adjusted, 30-day unplanned readmission, as per the HRRP. Independent variables were race (black, white), time (pre-HRRP, anticipation, penalty), and hospital penalty status (penalized, not penalized). Covariates included age, sex, and up to 9 comorbidities.^28^ Each target condition had distinct sets of comorbidities that were similar to those Medicare used to estimate expected readmission rates under the HRRP.^27^

### Statistical Analysis

First, we performed descriptive analyses of patient characteristics, using unpaired, 2-tailed *t* tests with SEs clustered at the hospital level to compare racial groups. Next, we compared hospital characteristics between penalized and nonpenalized hospitals using χ^2^ and *t* tests.

Third, we used linear probability regression models with hospital-level fixed effects to test whether mortality and readmission rates changed among black and white patients at any point after the HRRP was announced in 2010, and whether changes in these outcomes differed by race. When using hospital fixed effects, linear probability models are computationally less intensive than nonlinear models. Hospital fixed effects reduce the risk of confounding from measurable and unmeasurable time-invariant differences between hospitals.

Specifically, we estimated changes in outcomes in each quarter of the anticipation and penalty periods relative to a linear pre-HRRP time trend, allowing pre-HRRP trends (slope) and any changes in outcomes (intercept) to differ by race. Models controlled for patient characteristics, season, and hospital fixed effects. We calculated cluster-robust SEs at the hospital level, allowing for the correlation of regression model errors within hospitals. The eMethods in the Supplement provide model specifications.

We displayed regression estimates graphically, plotting observed risk-adjusted outcomes in each period along with projections of what outcome rates would have been in the anticipation and penalty periods without the HRRP, based on population-specific trends in the pre-HRRP period. To test whether the slope or intercept of the trends in outcomes changed at any point after the pre-HRRP period, we used coefficients from the regression models to calculate mean differences between observed and projected values during the penalty period for each racial group. To determine whether trends diverged between racial groups within the same hospitals, we calculated the difference between black and white patients in observed-to-projected differences. Statistical testing was 2-sided using *P* < .05 to indicate statistical significance. Although changes in readmissions began during the anticipation period,^8^ we simplified reporting by focusing on outcomes in the penalty period. In secondary analyses, we allowed coefficients to vary by hospital penalty status and calculated difference-in-differences between black and white patients within penalized and nonpenalized hospitals.

We performed 2 sets of sensitivity analyses. To test whether results were affected by assumptions about pre-HRRP trends, we projected outcome rates in the penalty period based on mean values during the pre-HRRP period. To assess whether results were affected by the type of regression model, we examined whether observed-to-projected differences differed between probit models and similarly specified linear probability models; which required removing interaction terms and hospital fixed effects from the linear probability model (eMethods in the Supplement).

Analyses were performed in Stata, version 14 (StataCorp LLC).

## Results

From January 1, 2007, through November 30, 2014, black adults 65 years and older had 627 373 index discharges and white adults 65 years and older had 5 845 130 index discharges. Black patients were more likely than white patients to be younger (mean [SD] age, 77.8 [8.3] vs 80.5 [8.2] years; *P* < .001), women (60.5% vs 53.7%; *P* < .001), dually covered by Medicare and Medicaid (45.7% vs 17.2%; *P* < .001), and treated at a penalized hospital (acute myocardial infarction, 82.8%; heart failure, 83.8% and pneumonia, 82.6% vs 69.6%, 73.3%; and 71.7%; all *P* < .001) (Table 1; eTable 1 in the Supplement reports characteristics by time and target condition).

**Table 1.** Characteristics of Black and White Adults 65 Years and Older Discharged in 2007-2014 From Hospitals Subject to the Medicare Hospital Readmissions Reduction Program Fiscal Year 2013

Patient Characteristic^a^	Black Patients	White Patients	*P* Value for Difference^b^
Primary diagnosis, No.			
AMI	97 798	1 151 988	NA
HF	355 866	2 516 613	NA
Pneumonia	173 709	2 176 529	NA
Age, mean (SD), y	77.8 (8.3)	80.5 (8.2)	<.001
Women, %	60.5	53.7	<.001
Medicare and Medicaid dual eligibility, %	45.7	17.2	<.001
Treated at a hospital penalized in fiscal year 2013, %			
AMI	82.8	69.6	<.001
HF	83.8	73.3	<.001
Pneumonia	82.6	71.7	<.001

Abbreviations: AMI, acute myocardial infarction; HF, heart failure; NA, not applicable.

^a^ Patient characteristics are at the level of the hospitalization.

^b^ SEs clustered at the hospital level.

### Acute Myocardial Infarction

**Figure 1.**
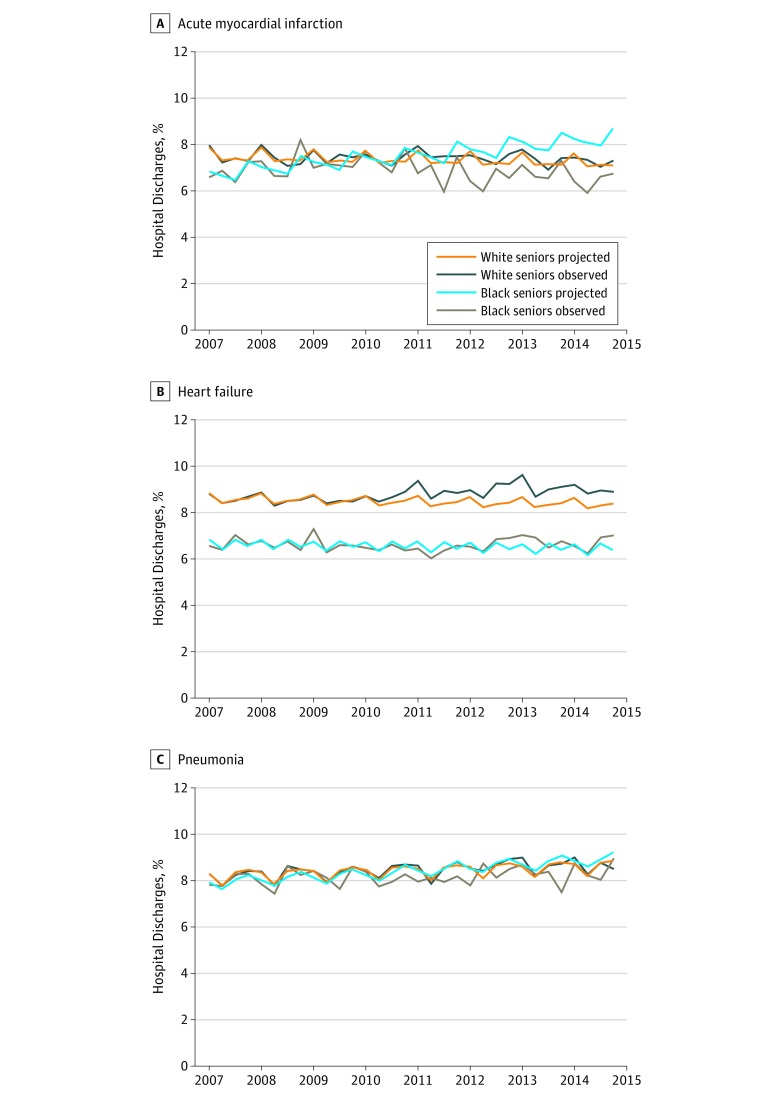
Risk-Adjusted 30-Day Postdischarge All-Cause Mortality Rates Among Black and White Adults 65 years and Older Observed mortality rates and rates projected based on trends in the pre-Medicare Hospital Readmissions Reduction Program period for acute myocardial infarction (A), heart failure (B), and pneumonia (C). Rates are shown by quarter year.

Risk-adjusted pre-HRRP mortality rates were 7.04% for black patients (95% CI, 6.75% to 7.33%) and 7.47% for white patients (95% CI, 7.37% to 7.57%) (eTable 2 in the Supplement). By the penalty period, the observed mortality rate had decreased relative to the projected rate among black patients (−1.52 percentage points, 95% CI, −3.02 to −0.02; *P* = .047). Among white patients, observed and projected rates were similar (0.13 points, 95% CI, −0.30 to 0.55; *P* = .561). Observed mortality decreased significantly more, relative to projections, among black than white patients within the same hospitals (difference-in-differences, −1.65 points; 95% CI, −3.19 to −0.10; *P* = .04) (Figure 1; eTable 2 in the Supplement).

**Figure 2.**
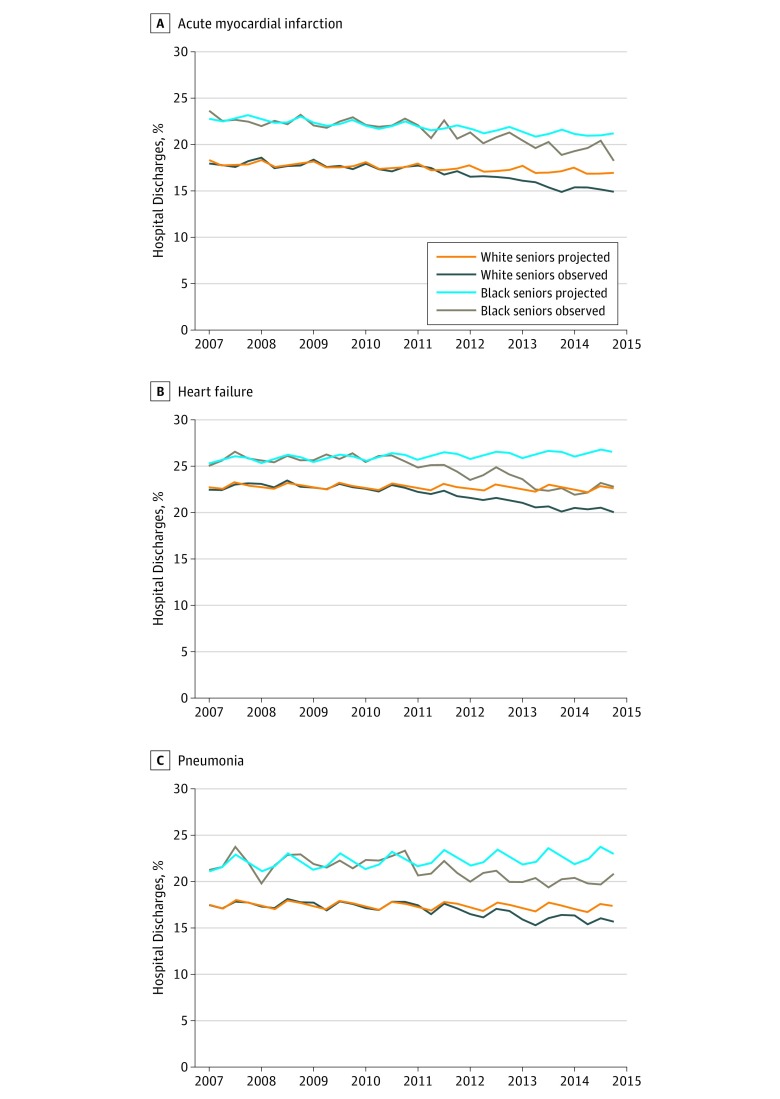
Risk-Adjusted 30-Day Unplanned Readmission Rates Among Black and White Adults 65 years and Older Observed readmission rates and rates projected based on trends in the pre-Medicare Hospital Readmissions Reduction Program period for acute myocardial infarction (A), heart failure (B), and pneumonia (C). Rates are shown by quarter year.

Despite the divergent trends in mortality, trends in readmissions were similar between the 2 groups (difference-in-differences, 0.18 points; 95% CI, −2.38 to 2.75; *P* = .89) (Figure 2; eTable 3 in the Supplement).

### Heart Failure

Risk-adjusted pre-HRRP mortality rates were 6.69% among black patients (95% CI, 6.56% to 6.82%) and 8.56% among white patients (95% CI, 8.48% to 8.64%). Among black patients, observed and projected mortality rates did not differ significantly (0.30 percentage points; 95% CI, −0.36 to 0.95; *P* = .37). However, observed mortality increased relative to projections among white patients (0.67 points; 95% CI, 0.35 to 0.98; *P* < .001). Observed-to-projected values were not significantly different between racial groups within the same hospitals (difference-in-differences, −0.37 points; 95% CI, −1.08 to 0.34; *P* = .31). Although mortality trends were similar, readmissions decreased more among black than white patients (difference-in-differences, −1.55 points; 95% CI, −3.02 to −0.08; *P* = .04).

### Pneumonia

Before the HRRP, risk-adjusted mortality rates were 8.08% for black patients (95% CI, 7.88% to 8.27%) and 8.27% for white patients (95% CI, 8.19% to 8.35%). Observed mortality rates during the penalty period were similar to projected rates among both black patients (−0.48 percentage points; 95% CI, −1.57 to 0.61; *P* = .39) and white patients (0.05 points; 95% CI, −0.27 to 0.38; *P* = .75). Observed-to-projected differences did not differ by race within hospitals (difference-in-differences, −0.54 points; 95% CI, −1.66 to 0.59; *P* = .35). Readmission trends did not differ significantly by race (difference-in-differences, −1.33 points; 95% CI, −3.08 to 0.42; *P* = .14).

### Results Stratified by Hospital Penalty Status

Of 3263 eligible hospitals, 2210 (67.7%) were penalized in fiscal year 2013. Penalized hospitals were larger, more often in urban areas, and treated higher percentages of black inpatients (Table 2).

**Table 2.** Characteristics of Hospitals Subject to the Medicare Hospital Readmissions Reduction Program in Fiscal Year 2013 or 2014, by Penalty Status in Fiscal Year 2013

Hospital Characteristic	Penalized Hospital^a^	Nonpenalized Hospital	*P* Value for Difference
Hospitals, No.^b^	2210	1053	
Hospital beds, mean (SD)	258 (232)	205 (217)	<.001
Location, %			
Urban	72.9	68.1	.005
Rural	27.1	31.9	
Medical school affiliation, %			
Yes	34.2	25.2	<.001
No	65.8	74.8	
Ownership status, %			
For-profit	21.6	18.4	.002
Nonprofit	61.0	59.7	
Government	17.1	21.1	
Physician ownership	0.2	0.8	
Index hospitalizations per year, mean (SD)^c^	270.3 (234.9)	209.2 (241.4)	<.001
% of patients who were black, mean (SD)^c^	12.0 (17.1)	7.3 (13.7)	<.001

^a^ Penalty status based was on penalties that Medicare assigned in fiscal year 2013 list (ie, 25 hospitals that appeared in fiscal year 2014 but not fiscal year 2013 were classified as nonpenalized). We lacked data on beds, location, medical school affiliation, and ownership status for 3 nonpenalized hospitals.

^b^ Hospitals were listed in the HRRP payment adjustment factor data sets reported by Medicare for fiscal years 2013 and 2014, with index hospitalizations that were eligible as per our analysis.

^c^ Based on data for myocardial infarction, heart failure, and pneumonia together from calendar years 2007-2014 (excluding December 2014).

**Figure 3.**
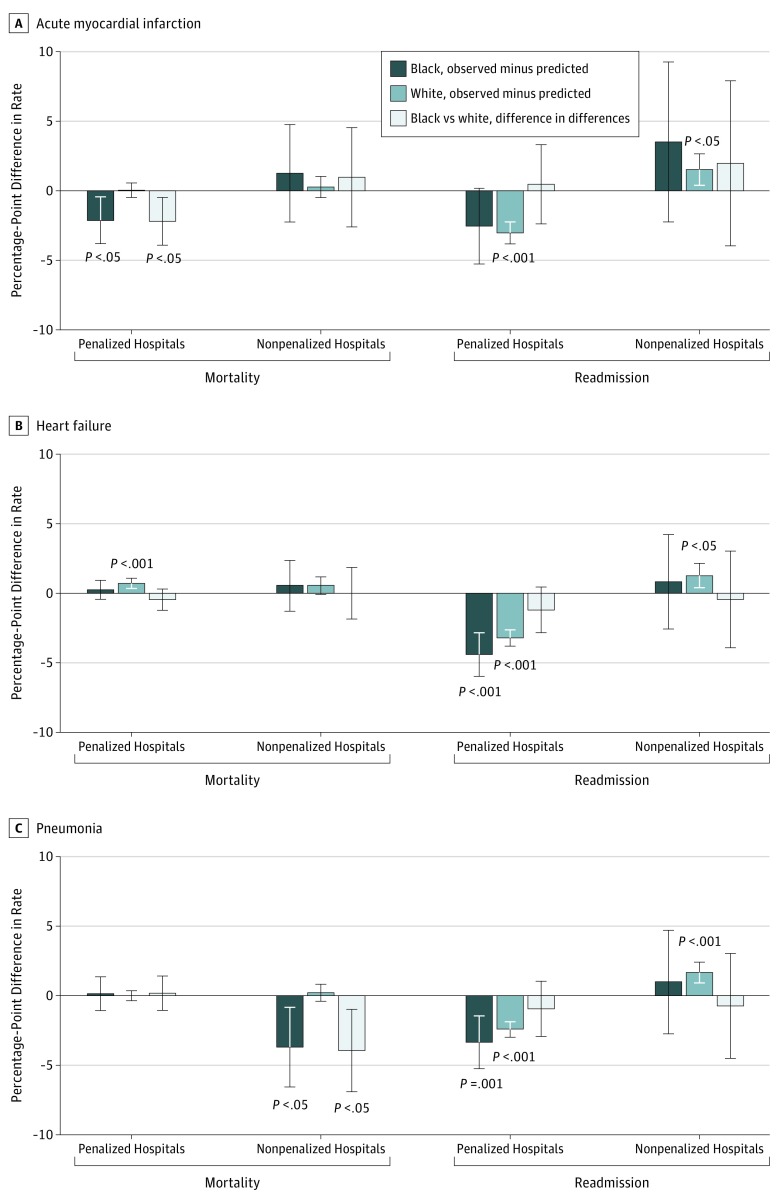
Difference Between Observed and Projected Risk-Adjusted 30-Day Rates, Stratified by Hospital Penalty Status Postdischarge all-cause mortality rates (A) and unplanned readmission rates (B) among black and white adults 65 years and older. Error bars represent 95% CIs.

Figure 3 and eTable 2 in the Supplement report differences between black and white patients in mortality trends at penalized and nonpenalized hospitals (difference-in-differences). They also show differences between observed and projected mortality for each racial group. Readmissions are shown for comparison (Figure 3; eTable 3 in the Supplement).

#### Acute Myocardial Infarction

For black patients, observed mortality decreased below projections at penalized hospitals (−2.11 percentage points; 95% CI, −3.76 to −0.46; *P* = .01), but not at nonpenalized hospitals (1.28 points; 95% CI, −2.21 to 4.78; *P* = .47). Among white patients, observed mortality was similar to projections at both penalized (0.06 points; 95% CI, −0.46 to 0.58; *P* = .82) and nonpenalized (0.29 points; 95% CI, −0.47 to 1.05; *P* = .46) hospitals.

Within penalized hospitals, mortality trends improved more among black patients than white patients (difference-in-differences, −2.17 points; 95% CI, −3.88 to −0.46; *P* = .01). Within nonpenalized hospitals, mortality trends were similar between the 2 groups (difference-in-differences, 0.99 points; 95% CI, −2.57 to 4.56; *P* = .59).

#### Heart Failure

For black patients, observed mortality did not differ significantly from projected mortality at either penalized (0.25 points; 95% CI, −0.44 to 0.95; *P* = .48) or nonpenalized (0.55 points; 95% CI, −1.27 to 2.37; *P* = .56) hospitals. Among white patients, observed mortality increased relative to projections at penalized hospitals (0.71 points; 95% CI, 0.35 to 1.07; *P* < .001), whereas changes were not significant at nonpenalized hospitals (0.55 points; 95% CI, −0.08 to 1.18; *P* = .09).

Trends were similar between black and white patients within penalized hospitals (difference-in-differences, −0.46 points; 95% CI, −1.22 to 0.31; *P* = .24) and within nonpenalized hospitals (difference-in-differences, 0.00 points; 95% CI, −1.87 to 1.88; *P* = .10).

#### Pneumonia

For black patients, observed and projected mortality were similar at penalized hospitals (0.18 percentage points; 95% CI, −1.00 to 1.36; *P* = .76), whereas observed mortality decreased relative to projections at nonpenalized hospitals (−3.67 points; 95% CI, −6.51 to −0.83; *P* = .01). Among white patients, mortality was similar to projections at penalized hospitals (−0.02 points; 95% CI, −0.41 to 0.37; *P* = .92) and nonpenalized hospitals (0.24 points; 95% CI, −0.35 to 0.83; *P* = .42).

Within penalized hospitals, trends were similar between the 2 groups (difference-in-differences, 0.20 points; 95% CI, −1.02 to 1.42; *P* = .74). Within nonpenalized hospitals, observed mortality decreased more, relative to projections, among black than white patients (difference-in-differences, −3.91 points; 95% CI, −6.84 to −0.98: *P* = .01).

### Sensitivity Analyses

Basing projected rates on pre-HRRP means instead of trends, mortality and readmission trends did not rise more or decreased less among black than white patients in the main or stratified analyses (eTable 2 and eTable 3 in the Supplement). Similarly specified probit and linear probability models yielded comparable results regarding mortality trends (eTable 4 in the Supplement). For example, in models stratified by race, we found similar increases in postdischarge mortality for white patients during the penalty period using a linear probability model (0.72 percentage points; 95% CI, 0.44-0.99; *P* < .001) and using a probit model (0.65 percentage points; 95% CI, 0.37-0.92; *P* < .001).

## Discussion

We found no evidence that 30-day postdischarge mortality worsened among black adults 65 years and older after implementation of the Medicare HRRP, either in general or relative to white adults 65 years and older treated at the same hospitals. Rather, AMI mortality trends improved among black patients, particularly in comparison with white patients, even though trends in AMI readmissions did not differ by race. For HF, mortality trends were stable among black patients, despite 2-fold larger decreases in HF readmissions among black patients and increases in HF mortality among white patients at the same hospitals. For pneumonia, mortality trends were stable and readmissions decreased in parallel for both racial groups. Decreases in mortality were larger among black patients than white patients with AMI within penalized hospitals and with pneumonia within nonpenalized hospitals.

These findings reveal that the post-HRRP changes in mortality reported by prior authors have not been uniformly distributed. Khera et al^13^ found upward trends in 30-day postdischarge mortality for HF and pneumonia that started before 2010 and continued unchanged thereafter. In contrast, Wadhera et al^14^ reported that trends in 30-day postdischarge mortality shifted, with decreases for AMI and increases for HF and pneumonia, after 2010. Several other analyses have also documented increases in 30-day postdischarge mortality for HF, relative to pre-HRRP trends.^11,12,15^ Our results indicate that decreases in AMI mortality were concentrated among black patients, while increases in HF mortality were limited to white patients.

The improved mortality for black patients with AMI is unexpected, in part because black patients had lower mortality rates than white patients at baseline. The mechanism by which mortality decreased is uncertain, including whether it was caused by the HRRP. In fiscal year 2014, the Medicare Value-Based Payment program started to incentivize improvements in 30-day mortality for AMI, HF, and pneumonia.^29^ We found, however, that decreases in AMI mortality among black patients started several years earlier. Moreover, many hospitals have reported enhancing transition-related care in response to the HRRP, which could have lowered both readmissions and mortality.^30,31,32,33,34^ Dharmarajan et al^11^ found that decreases in readmissions after 2010 were correlated at the hospital level with decreases in mortality. Improvements in transition-related care were less intensive at safety-net hospitals, where black patients are often admitted.^10,35^ Nonetheless, because black patients frequently face greater challenges navigating discharge transitions, quality improvement interventions might have been more effective for black than white populations within the same hospitals.^21^

Several factors suggest that contemporaneous events, rather than the HRRP, may be associated with increases in HF mortality rates among white patients. In prior research, changes in HF mortality were nearly identical at HRRP penalized and nonpenalized hospitals, despite much larger decreases in readmissions at penalized hospitals.^15^ This finding suggests that neither penalties nor decreasing readmission rates explain the increasing mortality. Instead, increasing mortality rates could reflect a sicker inpatient population. Other Medicare policies, such as the Two Midnight Rule, have incentivized shifts of lower-acuity inpatient care to outpatient settings.^36^ As such, rates of hospitalization have decreased, while measures of comorbidity among inpatients have risen.^13^ In addition, irrespective of setting, age-adjusted HF mortality rates increased from 2012 to 2014 and more deaths were from noncardiovascular causes.^37^

Prior research has also assessed the implications of value-based payment for black populations. In the Premier pay-for-performance program, 260 hospitals voluntarily provided data to the Centers for Medicare & Medicaid Services on 31 process-related quality measures and 2 outcome measures for AMI, HF, and pneumonia, as well as 3 surgical procedures. Hospitals in the 2 top deciles of performance received bonuses equal to 1% to 2% of Medicare payments, while hospitals in the 2 bottom deciles incurred penalties. Black patients with AMI and HF had lower baseline performance and exhibited greater improvements than white patients did.^38^ However, hospitals with higher percentages of socioeconomically disadvantaged patients had worse baseline performance and exhibited smaller improvements, leading to more financial penalties and fewer bonuses.^39^ Thus, both the Premier program and the HRRP improved performance among black patients while simultaneously placing greater financial stress on hospitals that treat socioeconomically disadvantaged populations.^10,40^

Because socioeconomic factors, including income and insurance, account for a substantial portion of racial differences in health outcomes,^41^ Medicare recently modified the HRRP. Starting in fiscal year 2019, performance will be assessed relative to other hospitals that admit similar percentages of patients who are dually eligible for Medicare and Medicaid.^7^ Given that 18% of dually eligible patients are black, compared with 7% of nondually eligible patients,^42^ some hospitals that care for larger numbers of black patients could see reduced penalties.

### Limitations

This study has limitations. As in prior studies, hospitals subject to the policy have no optimal control group, given that private payers and state governments have also instituted programs to reduce readmissions and hospitals exempt from the policy differ meaningfully from eligible hospitals.^2^ Shifts over time in rates of hospitalization, inpatient mortality, coded severity of illness, and length of stay could create the appearance of changes in mortality,^13,28,43^ but only if the shifts differed between black and white adults 65 years and older at the same hospitals. We did not assess mortality beyond 30 days.^44^ We included patients receiving hospice care, as per the HRRP.^7^ Future research should consider other measures that may be important to black adults 65 years and older affected by the HRRP, such as greater burdens to family caregivers.

## Conclusions

Postdischarge mortality did not appear to worsen for black adults 65 years and older under the HRRP, suggesting that certain value-based payment policies can be implemented without harming black populations. However, mortality seemed to worsen for white adults 65 years and older with HF and this warrants investigation.

## Notes

Supplement. **eMethods.** Details on Statistical Approach **eTable 1.** Characteristics of Black and White Seniors Discharged in 2007-2014 From Hospitals Subject to the Medicare Hospital Readmissions Reduction Program in Fiscal Year 2013, by Time Period **eTable 2.** Risk-Adjusted 30-Day Post-Discharge All-Cause Mortality Rates Among Black and White Seniors **eTable 3.** Risk-Adjusted 30-Day Unplanned Readmission Rates Among Black and White Seniors **eTable 4.** Percentage Point Differences Between Observed and Projected Mortality Rates During the HRRP Penalty Period Using Alternative Model Specifications, Stratified Estimates for Black and White Seniors
