# Supplementary material for: Thirty-Day Postdischarge Mortality Among Black and White Patients 65 Years and Older in the Medicare Hospital Readmissions Reduction Program
Source: JAMA Netw Open. 2019 Mar 15;2(3):e190634. doi: 10.1001/jamanetworkopen.2019.0634 (PMC6484660; doi:10.1001/jamanetworkopen.2019.0634)
Supplement: Supplement. — eMethods. Details on Statistical Approach eTable 1. Characteristics of Black and White Seniors Discharged in 2007-2014 From Hospitals Subject to the Medicare Hospital Readmissions Reduction Program in Fiscal Year 2013, by Time Period eTable 2. Risk-Adjusted 30-Day Post-Discharge All-Cause Mortality Rates Among Black and White Seniors eTable 3. Risk-Adjusted 30-Day Unplanned Readmission Rates Among Black and White Seniors eTable 4. Percentage Point Differences Between Observed and Projected Mortality Rates During the HRRP Penalty Period Using Alternative Model Specifications, Stratified Estimates for Black and White Seniors [file jamanetwopen-2-e190634-s001.pdf]

Supplementary Online Content

Huckfeldt P, Escarce J, Sood N, Yang Z, Popescu I, Nuckols T. Thirty-day postdischarge mortality among black and white patients 65 years and older in the Medicare Hospital Readmissions Reduction Program. *JAMA Netw Open*. 2019;2(3):e190634. doi:10.1001/jamanetworkopen.2019.0634

eMethods. Details on Statistical Approach

eTable 1. Characteristics of Black and White Seniors Discharged in 2007-2014 From Hospitals Subject to the Medicare Hospital Readmissions Reduction Program in Fiscal Year 2013, by Time Period

eTable 2. Risk-Adjusted 30-Day Post-Discharge All-Cause Mortality Rates Among Black and White Seniors

eTable 3. Risk-Adjusted 30-Day Unplanned Readmission Rates Among Black and White Seniors

eTable 4. Percentage Point Differences Between Observed and Projected Mortality Rates During the HRRP Penalty Period Using Alternative Model Specifications, Stratified Estimates for Black and White Seniors

This supplementary material has been provided by the authors to give readers additional information about their work.

© 2019 Huckfeldt P et al. *JAMA Network Open.*

eMethods. Details on Statistical Approach

Specification of regression model

We estimated linear interrupted time series models that indicated deviations from baseline time trends in each quarter of the anticipation and implementation periods and whether deviations differedbetween black and white seniors.

The main specification is shown in equation (1) below:

$$y_{iht} = \alpha + \rho_1 Quarter_t + \rho_2(Quarter_t \times Race_i) + \beta_0^q Season_t^q + \gamma Race_i + \delta X_{it} + \eta_h$$
$$+ \sum_{k=1}^{10} \lambda_0^k Anticipate_k + \sum_{k=1}^{9} \varphi_0^k Post_k + \beta^q (Season_t^q \times Race_i)$$
$$+ \Sigma_{k=1}^{10} \lambda^k (Anticipate_k \times Race_i) + \Sigma_{k=1}^{9} \varphi^k (Post_k \times Race_i) + u_{iht} \quad (1)$$

where y_{iht} is an outcome for patient *i* seen in hospital *h* in calendar quarter *t*; $Quarter_t$ is a linear time trend in calendar quarters ($Quarter_t$ = 1,2,3,…,32); $Season_q$ are binary indicator variables for the seasons of the year, to account for seasonality in hospitalization patterns and hospital outcomes; $Race_i$ is a binary indicator variable that indicates whether patient *i* is black; X_{it} are patient-level control variables (age, gender, and comorbidities); η_h are fixed effects for each hospital *h*, to absorb time-invariant hospital attributes; $Anticipate_1$ through $Anticipate_{10}$ are binary indicator variables representing each of 10 calendar quarters in the anticipation period (April 1, 2010-September 30, 2012); $Post_1$ through $Post_9$ are binary indicator variables representing each of 9 quarters in the implementation period (October 1, 2012-November 30, 2014); and $Quarter_t \times Race_i$, $Season_q \times Race_i$, $Anticipate_k \times Race_i$, and $Post_k \times Race_i$ are interaction terms. The estimates of interest include λ^1 through λ^{10} and φ^1 through φ^9, the coefficients of the interaction terms, which express the relative change in the outcome *y* in each quarter of the anticipation or implementation period for black versus white seniors.

Testing sensitivity of results to use of linear model specification

We used linear probability models to estimate the association of HRRP with mortality and how this varies by race. One potential concern is that 30-day post-discharge mortality rates are below 10% and thus linear probability models could lead to predicted probabilities below zero and thus differ from probit or logistic model estimates where predicted values are bound between 0 and 1. To account for this, we explored the sensitivity of our estimates to using alternative nonlinear model specifications. It was not feasible to exactly replicate our main estimation using probit or logistic models, because: (1) interaction terms such as those we include between race and time periods in our main models have a complex interpretation in nonlinear models that differs from linear models and the *sign* of interaction effects may vary across different values of covariates[1] and (2) we include hospital fixed effects that are computationally intensive to estimate in a nonlinear context and may lead to nonconvergence of model estimates. Instead, we estimated separate stratified interrupted time series models by race, which avoided the need to estimate and interpret interaction terms. We estimated linear probability models with hospital fixed effects, linear probability models without hospital fixed effects, and probit models without hospital fixed effects in order to determine the sensitivity of our models to both the inclusion of hospital fixed effects and then the sensitivity to using a linear specification. For the probit models, we calculated the difference between observed versus predicted mortality after HRRP implementation using the sample average of marginal effects and, separately, calculating marginal effects at the average of each regressor. The resulting post-HRRP estimates are displayed in eTable 4.

For AMI, we found statistically insignificant estimates of slightly varying magnitudes across the four models for both black and white seniors. For HF, we found marginal effects across the four models for black and white seniors; estimates for black seniors were all statistically insignificant while estimates

[1] Ai, C. and E. C. Norton (2003). "Interaction Terms in Logit and Probit Models." Economics Letters 80(1): 123-129

© 2019 Huckfeldt P et al. *JAMA Network Open.*

for white seniors were all statistically significant. For pneumonia, we found slightly varying and all statistically insignificant estimates across the four models for black seniors. For white seniors, we found very similar estimates across the four models that were all statistically significant (or borderline significant in one case). We concluded that our results were not sensitive to the use of a linear rather than nonlinear specification.

© 2019 Huckfeldt P et al. *JAMA Network Open.*

eTable 1.
Characteristics of Black and White Seniors Discharged in 2007-2014 From Hospitals Subject to the Medicare Hospital Readmissions Reduction Program in Fiscal Year 2013, by Time Period

Patient Characteristics		Black Seniors			White Seniors		
		Pre-HRRP*	Anticipation	Penalty	Pre-HRRP	Anticipation	Penalty
Primary Diagnosis, N							
	AMI	40,830	30,766	26,202	500,480	354,243	297,265
	HF	155,052	110,985	89,829	1,110,712	773,466	632,435
	Pneumonia	76,373	53,894	43,442	979,649	669,441	527,439
Age, Mean (SD)		77.8 (8.3)	77.9 (8.3)	77.7 (8.4)	80.4 (8.0)	80.6 (8.2)	80.5 (8.4)
Male, N (%)		107,053 (39.3)	77,161 (39.4)	63,800 (40.0)	1,186,337 (45.8)	832,161 (46.3)	686,993 (47.2)
Medicaid Dual Eligibility, N (%)		126,213 (46.4)	90,188 (46.1)	70,477 (44.2)	447,299 (17.3)	312,839 (17.4)	244,209 (16.8)
Comorbidities, Mean (SD)		2.1 (1.2)	2.0 (1.1)	2.0 (1.1)	2.2 (1.2)	2.1 (1.1)	2.0 (1.1)
Treated at a Hospital Penalized in Fiscal Year 2013, N (%)							
	AMI	33,915 (83.1)	25,493 (82.9)	21,593 (82.4)	350,319 (70.0)	246,652 (69.6)	205,074 (69.0)
	HF	130,719 (84.3)	93,008 (83.8)	74,556 (83.0)	820,993 (73.9)	567,739 (73.4)	455,424 (72.0)
	Pneumonia	63,359 (83.0)	44,588 (82.7)	35,515 (81.8)	703,580 (71.8)	480,574 (71.8)	375,342 (71.2)
Unadjusted 30-Day All-Cause Mortality Rate, Mean (SD)							
	AMI	7.52 (26.4)	6.99 (25.5)	6.67 (25.0)	7.58 (26.5)	7.42 (26.2)	7.14 (25.8)
	HF	5.18 (22.2)	5.45 (22.7)	6.02 (23.8)	8.30 (27.6)	9.18 (28.9)	9.80 (29.7)
	Pneumonia	7.93 (27.0)	8.25 (27.5)	8.33 (27.6)	8.06 (27.2)	8.67 (28.1)	8.88 (28.5)
Unadjusted 30-Day Unplanned Readmission Rate, Mean (SD)							
	AMI	23.10 (42.1)	21.88 (41.3)	20.12 (40.1)	18.03 (38.4)	16.98 (37.5)	15.25 (36.0)
	HF	25.90 (43.8)	25.21 (43.4)	23.14 (42.2)	22.71 (41.9)	22.04 (41.5)	20.60 (40.4)
	Pneumonia	21.58 (41.1)	21.39 (41.0)	20.02 (40.0)	17.44 (37.9)	17.10 (37.6)	16.03 36.7)

© 2019 Huckfeldt P et al. *JAMA Network Open.*

Abbreviations: HRRP = Medicare Hospital Readmissions Reduction Program, N = number, SD = standard deviation, AMI = acute myocardial infarction, HF = heart failure.

*The pre-HRRP period was January 2007 to March 2010. The anticipation period was April 2010 to September 2012, after the Affordable Care Act was passed and before the HRRP was implemented. The HRRP penalty period was October 2012 to November 2014, when eligible hospitals were potentially subject to HRRP penalties.

© 2019 Huckfeldt P et al. *JAMA Network Open.*

eTable 2:
Risk-Adjusted 30-Day Post-Discharge All-Cause Mortality Rates Among Black and White Seniors

	AMI	HF	Pneumonia
Hospitals, Total	3,158	3,228	3,250
Index Hospitalizations, Total	1,249,786	2,872,479	2,350,238
**Risk-Adjusted Rates in Pre-HRRP Period, Mean (95% CI)**			
Black Seniors	7.04 (6.75 to 7.33)	6.69 (6.56 to 6.82)	8.08 (7.88 to 8.27)
White Seniors	7.47 (7.37 to 7.57)	8.56 (8.48 to 8.64)	8.27 (8.19 to 8.35)
**Risk-Adjusted Trends (Slope) in Pre-HRRP Period, Mean (95% CI)**			
Black Seniors	0.05 (-0.02 to 0.12)	-0.01 (-0.04 to 0.02)	0.03 (-0.01 to 0.08)
White Seniors	-0.01 (-0.03 to 0.01)	-0.01 (-0.02 to 0.01)	0.02 (0.00 to 0.03)
**Differences Between Observed and Projected Rates within Each Racial Group and Between Groups (Diff-in-diff) During the Penalty Period**			
**Percentage Point Differences When Projected Rates Are Based on Trends in the Pre-HRRP Period, Mean (95% CI), P-value**			
Black Seniors (Difference)	-1.52 (-3.02 to -0.02), P=0.047	0.30 (-0.36 to 0.95), P=0.371	-0.48 (-1.57 to 0.61), P=0.387
White Seniors (Difference)	0.13 (-0.30 to 0.55), P=0.561	0.67 (0.35 to 0.98), P<0.001	0.05 (-0.27 to 0.38), P=0.748
Black vs. White Seniors (Diff-in-diff)	-1.65 (-3.19 to -0.10), P=0.037	-0.37 (-1.08 to 0.34), P=0.305	-0.54 (-1.66 to 0.59), P=0.352
Stratified by Hospital Penalty Status			
Penalized Hospitals			
Black Seniors (Difference)	-2.11 (-3.76 to -0.46), P=0.012	0.25 (-0.44 to 0.95), P=0.478	0.18 (-1.00 to 1.36), P=0.761
White Seniors (Difference)	0.06 (-0.46 to 0.58), P=0.821	0.71 (0.35 to 1.07), P<0.001	-0.02 (-0.41 to 0.37), P=0.915
Black vs. White Seniors (Diff-in-diff)	-2.17 (-3.88 to -0.46), P=0.013	-0.46 (-1.22 to 0.31), P=0.244	0.20 (-1.02 to 1.42), P=0.743
Non-Penalized Hospitals			
Black Seniors (Difference)	1.28 (-2.21 to 4.78), P=0.472	0.55 (-1.27 to 2.37), P=0.555	-3.67 (-6.51 to -0.83), P=0.011
White Seniors (Difference)	0.29 (-0.47 to 1.05), P=0.457	0.55 (-0.08 to 1.18), P=0.089	0.24 (-0.35 to 0.83), P=0.420
Black vs. White Seniors (Diff-in-diff)	0.99 (-2.57 to 4.56), P=0.585	0.00 (-1.87 to 1.88), P=0.998	-3.91 (-6.84 to -0.98), P=0.009
**Percentage Point Differences When Projected Rates Are Based on Mean Rates in the Pre-HRRP Period, Mean (95% CI), P-value**			
Black Seniors (Difference)	-0.48 (-0.88 to -0.07), P=0.020	0.11 (-0.09 to 0.31), P=0.279	0.23 (-0.12 to 0.58), P=0.204
White Seniors (Difference)	-0.04 (-0.16 to 0.09), P=0.554	0.50 (0.40 to 0.60), P<0.001	0.38 (0.27 to 0.50), P<0.001
Black vs. White Seniors (Diff-in-diff)	-0.44 (-0.86 to -0.02), P=0.040	-0.39 (-0.61 to -0.17), P<0.001	-0.16 (-0.51 to 0.20), P=0.394
Stratified by Hospital Penalty Status			
Penalized Hospitals			

© 2019 Huckfeldt P et al. *JAMA Network Open.*

		Black Seniors (Difference)	-0.47 (-0.92 to -0.02), P=0.040	0.12 (-0.10 to 0.33), P=0.285	0.39 (0.01 to 0.78), P=0.045
		White Seniors (Difference)	-0.06 (-0.21 to 0.10), P=0.468	0.46 (0.35 to 0.58), P<0.001	0.38 (0.25 to 0.52), P<0.001
		Black vs. White Seniors (Diff-in-diff)	-0.41 (-0.89 to 0.06), P=0.086	-0.35 (-0.58 to -0.11), P=0.004	0.01 (-0.38 to 0.41), P=0.952
	Non-Penalized Hospitals				
		Black Seniors (Difference)	-0.55 (-1.48 to 0.39), P=0.251	0.08 (-0.41 to 0.57), P=0.751	-0.55 (-1.38 to 0.27), P=0.190
		White Seniors (Difference)	0.01 (-0.21 to 0.22), P=0.940	0.59 (0.38 to 0.79), P<0.001	0.39 (0.18 to 0.59), P<0.001
		Black vs. White Seniors (Diff-in-diff)	-0.56 (-1.51 to 0.40), P=0.254	-0.51 (-1.03 to 0.02), P=0.059	-0.94 (-1.78 to -0.10), P=0.029

Abbreviations: HRRP = Medicare Hospital Readmissions Reduction Program, 95% CI = 95% confidence interval, AMI = acute myocardial infarction, HF = heart failure.

Notes: “Difference” indicates difference between observed and projected values; “Diff-in-diff” indicates difference between black and white patients in observed vs. projected values (difference in differences).

© 2019 Huckfeldt P et al. *JAMA Network Open.*

eTable 3: Risk-Adjusted 30-Day Unplanned Readmission Rates Among Black and White Seniors

	AMI	HF	Pneumonia
Hospitals, Total	3,158	3,228	3,250
Index Hospitalizations, Total	1,249,786	2,872,479	2,350,238
**Risk-Adjusted Rates in Pre-HRRP Period, Mean (95% CI)**			
Black Seniors	22.37 (21.88 to 22.86)	25.66 (25.32 to 25.99)	21.71 (21.34 to 22.08)
White Seniors	17.88 (17.69 to 18.06)	22.79 (22.64 to 22.95)	17.47 (17.33 to 17.60)
**Risk-Adjusted Trends (Slope) in Pre-HRRP Period, Mean (95% CI)**			
Black Seniors	-0.06 (-0.17 to 0.05)	0.04 (-0.03 to 0.10)	0.04 (-0.03 to 0.12)
White Seniors	-0.03 (-0.06 to 0.00)	-0.00 (-0.03 to 0.02)	-0.01 (-0.03 to 0.01)
**Differences Between Observed and Projected Rates within Each Racial Group and Between Groups (Diff-in-Diff) During the Penalty Period**			
**Percentage Point Differences When Projected Rates Are Based on Trends in the Pre-HRRP Period, Mean (95% CI), P-value**			
Black Seniors (Difference)	-1.45 (-3.91 to 1.01), P=0.249	-3.57 (-4.98 to -2.16), P<0.001	-2.57 (-4.26 to -0.89), P=0.003
White Seniors (Difference)	-1.63 (-2.29 to -0.97), P<0.001	-2.02 (-2.51 to -1.53), P<0.001	-1.24 (-1.70 to -0.78), P<0.001
Black vs. White Seniors (Diff-in-diff)	0.18 (-2.38 to 2.75), P=0.889	-1.55 (-3.02 to -0.08), P=0.039	-1.33 (-3.08 to 0.42), P=0.136
Stratified by Hospital Penalty Status			
Penalized Hospitals			
Black Seniors (Difference)	-2.51 (-5.23 to 0.21), P=0.071	-4.41 (-5.96 to -2.87), P<0.001	-3.32 (-5.20 to -1.44), P=0.001
White Seniors (Difference)	-3.00 (-3.79 to -2.21), P<0.001	-3.21 (-3.79 to -2.62), P<0.001	-2.40 (-2.96 to -1.83), P<0.001
Black vs. White Seniors (Diff-in-diff)	0.49 (-2.35 to 3.34), P=0.735	-1.21 (-2.84 to 0.42), P=0.145	-0.92 (-2.89 to 1.04), P=0.358
Non-Penalized Hospitals			
Black Seniors (Difference)	3.53 (-2.20 to 9.25), P=0.227	0.83 (-2.57 to 4.23), P=0.632	0.98 (-2.73 to 4.70), P=0.604
White Seniors (Difference)	1.54 (0.41 to 2.66), P=0.007	1.28 (0.42 to 2.13), P=0.003	1.69 (0.96 to 2.42), P<0.001
Black vs. White Seniors (Diff-in-diff)	1.99 (-3.93 to 7.91), P=0.510	-0.44 (-3.91 to 3.02), P=0.801	-0.71 (-4.49 to 3.07), P=0.712
**Percentage Point Differences When Projected Rates Are Based on Mean Rates in the Pre-HRRP Period, Mean (95% CI), P-value**			
Black Seniors (Difference)	-2.66 (-3.35 to -1.98), P<0.001	-2.83 (-3.23 to -2.42), P<0.001	-1.71 (-2.25 to -1.17), P<0.001
White Seniors (Difference)	-2.19 (-2.38 to -2.00), P<0.001	-2.11 (-2.28 to -1.94), P<0.001	-1.51 (-1.67 to -1.36), P<0.001
Black vs. White Seniors (Diff-in-diff)	-0.47 (-1.18 to 0.24), P=0.194	-0.72 (-1.14 to -0.30), P=0.001	-0.20 (-0.74 to 0.35), P=0.483
Stratified by Hospital Penalty Status			
Penalized Hospitals			

© 2019 Huckfeldt P et al. *JAMA Network Open.*

		Black Seniors (Difference)	-2.88 (-3.63 to -2.12), P<0.001	-3.08 (-3.53 to -2.63), P<0.001	-2.02 (-2.61 to -1.42), P<0.001
		White Seniors (Difference)	-2.62 (-2.85 to -2.39), P<0.001	-2.44 (-2.65 to -2.24), P<0.001	-1.80 (-1.99 to -1.62), P<0.001
		Black vs. White Seniors (Diff-in-diff)	-0.26 (-1.05 to 0.54), P=0.527	-0.64 (-1.11 to -0.17), P=0.008	-0.21 (-0.83 to 0.40), P=0.497
	Non-Penalized Hospitals				
		Black Seniors (Difference)	-1.65 (-3.21 to -0.08), P=0.039	-1.50 (-2.35 to -0.65), P=0.001	-0.24 (-1.43 to 0.95), P=0.687
		White Seniors (Difference)	-1.23 (-1.54 to -0.92), P<0.001	-1.22 (-1.48 to -0.95), P<0.001	-0.79 (-1.04 to -0.53), P<0.001
		Black vs. White Seniors (Diff-in-diff)	-0.42 (-2.00 to 1.17), P=0.606	-0.28 (-1.17 to 0.60), P=0.534	0.54 (-0.64 to 1.72), P=0.367

Abbreviations: HRRP = Medicare Hospital Readmissions Reduction Program, 95% CI = 95% confidence interval, AMI = acute myocardial infarction, HF = heart failure.

Notes: “Difference” indicates difference between observed and projected values; “Diff-in-diff” indicates difference between black and white patients in observed vs. projected values (difference in differences).

© 2019 Huckfeldt P et al. *JAMA Network Open.*

eTable 4. Percentage Point Differences Between Observed and Projected Mortality Rates During the HRRP Penalty Period Using Alternative Model Specifications, Stratified Estimates for Black and White Seniors

	**Linear Probability Model Estimates**		**Probit Estimates**	
	**With Hospital Fixed Effects**	**Without Hospital Fixed Effects**	**Based on Sample Mean of Marginal Effects**	**Based on Marginal Effects Calculated at Mean of Covariates**
**Percentage Point Differences When Projected Rates Are Based on Trends in the Pre-HRRP Period, Mean (95% CI), P-value**				
**AMI**				
Black seniors	-1.06 (-2.33 to 0.20), P=0.100	-1.00 (-2.26 to 0.26), P=0.120	-0.94 (-2.16 to 0.29), P=0.134	-0.83 (-1.92 to 0.26), P=0.134
White seniors	0.23 (-0.14 to 0.60), P=0.215	0.25 (-0.12 to 0.62), P=0.181	0.17 (-0.19 to 0.53), P=0.350	0.15 (-0.16 to 0.45), P=0.350
**HF**				
Black seniors	0.33 (-0.25 to 0.91), P=0.265	0.39 (-0.19 to 0.96), P=0.186	0.34 (-0.24 to 0.92), P=0.252	0.31 (-0.22 to 0.85) P=0.252
White seniors	0.72 (0.44 to 0.99), P<0.001	0.73 (0.45 to 1.00), P<0.001	0.65 (0.37 to 0.92), P<0.001	0.62 (0.36 to 0.89), P<0.001
**Pneumonia**				
Black seniors	-0.73 (-1.67 to 0.20), P=0.126	-0.59 (-1.53 to 0.34), P=0.213	-0.57 (-1.50 to 0.35), P=0.225	-0.53 (-1.39 to 0.33), P=0.225
White seniors	0.29 (0.01 to 0.56), P=0.044	0.27 (-0.01 to 0.55), P=0.057	0.29 (0.01 to 0.57), P=0.041	0.27 (0.01 to 0.54), P=0.041

Abbreviations: HRRP = Medicare Hospital Readmissions Reduction Program, 95% CI = 95% confidence interval, AMI = acute myocardial infarction, HF = heart failure.

© 2019 Huckfeldt P et al. *JAMA Network Open.*
